# An Optimized Fibril Network Morphology Enables High‐Efficiency and Ambient‐Stable Polymer Solar Cells

**DOI:** 10.1002/advs.202001986

**Published:** 2020-07-26

**Authors:** Jiali Song, Linglong Ye, Chao Li, Jinqiu Xu, Sreelakshmi Chandrabose, Kangkang Weng, Yunhao Cai, Yuanpeng Xie, Padraic O'Reilly, Kai Chen, Jiajia Zhou, Yi Zhou, Justin M. Hodgkiss, Feng Liu, Yanming Sun

**Affiliations:** ^1^ School of Chemistry Beihang University Beijing 100191 P. R. China; ^2^ Department of Polymer Science and Engineering School of Chemistry and Chemical Engineering Shanghai Jiao Tong University Shanghai 200240 P. R. China; ^3^ MacDiarmid Institute for Advanced Materials and Nanotechnology and School of Chemical and Physical Sciences Victoria University of Wellington Wellington 6010 New Zealand; ^4^ Molecular Vista Inc. 6840 Via Del Oro, Suite 110 San Jose CA 95119 USA; ^5^ Laboratory of Advanced Optoelectronic Materials College of Chemistry Chemical Engineering and Materials Science Soochow University Suzhou 215123 P. R. China

**Keywords:** copolymers, fibril network morphology, morphology control, organic solar cells, polymer solar cells

## Abstract

Morphological stability is crucially important for the long‐term stability of polymer solar cells (PSCs). Many high‐efficiency PSCs suffer from metastable morphology, resulting in severe device degradation. Here, a series of copolymers is developed by manipulating the content of chlorinated benzodithiophene‐4,8‐dione (T1‐Cl) via a random copolymerization approach. It is found that all the copolymers can self‐assemble into a fibril nanostructure in films. By altering the T1‐Cl content, the polymer crystallinity and fibril width can be effectively controlled. When blended with several nonfullerene acceptors, such as TTPTT‐4F, O‐INIC3, EH‐INIC3, and Y6, the optimized fibril interpenetrating morphology can not only favor charge transport, but also inhibit the unfavorable molecular diffusion and aggregation in active layers, leading to excellent morphological stability. The work demonstrates the importance of optimization of fibril network morphology in realizing high‐efficiency and ambient‐stable PSCs, and also provides new insights into the effect of chemical structure on the fibril network morphology and photovoltaic performance of PSCs.

## Graphical abstract

A series of copolymers via a random copolymerization approach are designed and synthesized. The well‐defined fibril interpenetrating morphology with appropriate phase separation in PT2‐based blends can efficiently suppress the unfavorable aggregation, resulting in excellent morphological stability and high efficiency. The work demonstrates the importance of optimization of fibril network morphology in realizing high‐efficiency and ambient‐stable polymer solar cells.

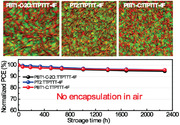

Polymer solar cells (PSCs) consisting of a mixture of a conjugated polymer donor and a small molecule acceptor (SMA) have attracted considerable attention due to their unique advantages, such as light weight, low cost, and flexibility.^[^ ^1^, ^2^, ^3^, ^4^ ^]^ With the recent emergence of nonfullerene acceptors (NFAs), the photovoltaic performance of PSCs undergoes a rapid improvement and the maximum power conversion efficiency (PCE) is already exceeding 16%.^[^ ^5^, ^6^, ^7^, ^8^, ^9^ ^]^ To achieve high PCEs, suitable donor–acceptor phase separation with a bicontinuous network morphology should be formed in order to ensure efficient exction dissociation and charge transport.^[^ ^10^, ^11^ ^]^ Besides, the morphology stability is crucially important for the long‐term stability of PSCs.^[^ ^12^, ^13^ ^]^ Many high‐efficiency PSCs suffer from metastable morphology,^[^ ^14^, ^15^ ^]^ and the molecule diffusion and aggregation could result in severe device degradation.^[^ ^16^, ^17^ ^]^

Recently, we have proposed a fibril network strategy (FNS) to control the bulk‐heterojunction (BHJ) morphology.^[^ ^10^, ^18^, ^19^ ^]^ We used polymer fibrils to form the hole transporting network and the SMA inserted in‐between the fibril network to transport electron, which is quite analogous to the ideal BHJ bicontinuous network. FNS has been successfully applied in fullerene, NFA, and multi‐component PSCs to control BHJ morphology.^[^ ^10^, ^20^, ^21^, ^22^, ^23^, ^24^, ^25^ ^]^ In this work, we mainly focus on investigating the relationship between fibril network nanostructure and morphology stability of PSCs, which has been rarely studied in the literature. Here, we specially designed and synthesized a series of copolymers via random copolymerization strategy using alkylphenyl substituted benzodithiophene (M1), benzodithiophene‐4,8‐dione (T1), and chlorinated T1 (T1‐Cl) as the original materials (**Figure** **1**a). Among them, PBT1‐C, and PBT1‐C‐2Cl were reported in our previous work.^[^ ^10^, ^24^ ^]^ Compared with PBT1‐C, PBT1‐C‐2Cl shows increased degree of crystallinity and a larger fibril width in film due to the interchain chalcogen–chalcogen interactions of chlorine atoms. By manipulating T1‐Cl contents, the crystallinity and fibril width of copolymers could be fine‐tuned. Among the copolymers, PT2 possesses the narrowest fibril width and moderate crystallinity. When blended with TTPTTT‐4F NFA,^[^ ^26^ ^]^ the optimized fibril network morphology in PT2:TTPTTT‐4F film led to superior long‐term stability of PSCs. The unencapsulated devices can maintain over 95% of its initial PCE after 2250 h. Besides the TTPTTT‐4F, PT2 can match well with other high‐performance NFAs, such as O‐INIC3,^[^ ^27^, ^28^ ^]^ EH‐INIC3, and Y6,^[^ ^29^ ^]^ and all the corresponding devices exhibited superior long‐term stability and high PCEs.

**Figure 1:**
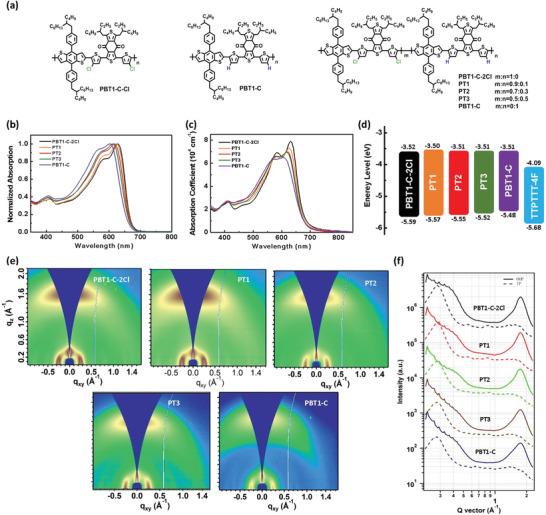
a) Chemical structures of PBT1‐C‐2Cl, PBT1‐C, and the copolymers. b,c) Absorption spectra of copolymers in chlorobenzene solution (b) and thin film (c). d) Energy level diagrams of copolymers and TTPTT‐4F. e) 2D GIWAXS patterns and f) the corresponding out‐of‐plane (solid line) and in‐plane (dashed line) line cuts of copolymer neat films.

UV–vis absorption spectra of the copolymers are shown in Figure 1b,c, and the summarized data are provided in Table S1, Supporting Information. Gradual blueshift in absorption was observed for copolymers from PBT1‐C‐Cl to PBT1‐C in both solution and thin film. The main absorption peak in the long wavelength was depressed, indicating decreased aggregation in the solid state with decreasing T1‐Cl content in copolymers.^[^ ^7^, ^30^ ^]^ The optical bandgaps (*E*_g_^opt^) estimated from the onset of optical absorption edge are 1.83, 1.83, 1.82, 1.82, and 1.81 eV for PBT1‐C‐Cl, PT1, PT2, PT3, and PBT1‐C, respectively. The energy levels were determined by cyclic voltammetry measurements (Figure S1, Supporting Information). The highest occupied molecular orbital (HOMO) and lowest unoccupied molecular orbital level were calculated to be −5.59/−3.52, −5.57/−3.50, −5.55/−3.51, −5.52/−3.51, and −5.48/−3.51 eV for PBT1‐C‐Cl, PT1, PT2, PT3, and PBT1‐C, respectively. The upshifted HOMO energy levels of the random copolymers are mainly ascribed to the reduced electron‐withdrawing effect of chlorine atoms as the amount of T1‐Cl decreased. Additionally, as shown in Figure 1d, all the copolymers feature well‐matched energy levels with TTPTTT‐4F, thus providing an affordable driving force for efficient exciton separation.^[^ ^31^ ^]^

The grazing‐incidence wide‐angle X‐ray scattering (GIWAXS) was adopted to study the crystallinity and molecular packing behavior of the neat copolymers. The 2D GIWAXS patterns and line‐cut profiles of neat polymers films are shown in Figure 1e,f, and the crystal coherence length (CCL) and d‐spacing distances are summarized in **Table** **1**. As illustrated in Table 1, all the copolymers display preferential formation of the face‐on orientation, as evidenced by strong (100) lamellar scattering peak in the in‐plane (IP) direction and (010) pronounced *π*–*π* stacking peak in the out‐of‐plane (OOP) direction.^[^ ^32^ ^]^ Moreover, from PBT1‐C‐Cl to PBT1‐C, the location of (010) diffraction peaks in the OOP direction shift from *q*_z_ = 1.76 to 1.73 Å^−1^, corresponding to gradually increased *π*–*π* stacking distance of 3.57 to 3.63 Å, and the intensity of (010) peaks are gradually reduced with the CCL values decreased from 15.55 to 11.25 Å. Meanwhile, a similar trend can be also observed for (100) lamellar diffraction peaks in the IP direction. These results indicate that the crystallinity of the copolymers could be effectively modulated by manipulating T1‐Cl content in the polymer backbone. The hole mobilities (*μ*_h_) measured by space charge limited current are 1.12 × 10^−3^, 1.00 × 10^−3^, 9.39 × 10^−4^, 8.77 × 10^−4^, and 8.14 × 10^−4^ cm^2^ V^−1^ s^−1^ for PBT1‐C‐2Cl, PT1, PT2, PT3, and PBT1‐C, respectively (Figure S2 and Table S2, Supporting Information), agreeing well with the GIWAXS results.

**Table 1:** Coherence lengths of the (100) and (010) peaks and the d‐spacings of PBT1‐C‐2Cl, PBT1‐C, and PT1‐PT3

Materials	(100) CL [Å]	(100) *d*‐spacing [Å]	(010) CL [Å]	(010) *d*‐spacing [Å]
PBT1‐C‐2Cl	70.19	22.39	15.55	3.57
PT1	71.68	22.41	15.16	3.58
PT2	63.96	22.63	13.34	3.60
PT3	58.44	22.70	12.70	3.61
PBT1‐C	57.67	22.97	11.25	3.63

Atomic force microscopy (AFM) was performed to investigate the polymer morphology. As shown in **Figure** **2**, all neat copolymers exhibit prominent fibril network morphology. In detailed comparison, the fibrils in PBT1‐C‐2Cl film are bound together, indicative of strong aggregation. When 10% T1‐Cl content is employed, the aggregation is effectively suppressed and uniform fibril morphology was formed. The results are consistent with the absorption and GIWAXS data. Further decreasing T1‐Cl contents in copolymers seems to be of no obvious effect on fibril morphology and the calculated fibril width through image analysis using Otsu thresholding^[^ ^33^ ^]^ was in the range of 14–16 nm. The PT2 neat film was found to show the narrowest fibril width (*d* = 14.4 nm), and the moderate crystallinity.

**Figure 2:**
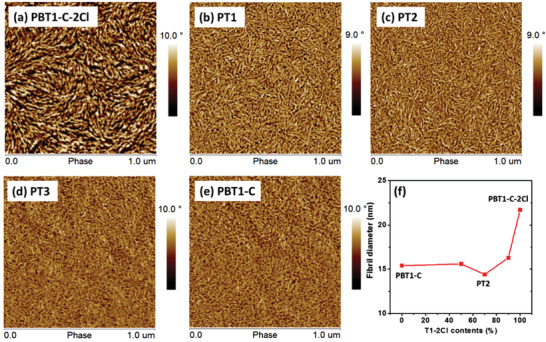
a–e) AFM phase images of copolymer neat films. f) Fibril diameter of copolymer neat films.

To evaluate the photovoltaic performance of these copolymers, PSCs with an inverted structure of indium tin oxide (ITO)/zinc oxide (ZnO)/active layer/molybdenum trioxide (MoO_3_)/silver (Ag) was fabricated. The asymmetric TTPTTT‐4F was used as the acceptor.^[^ ^26^ ^]^ The optimal active layers were obtained by spin‐coating the chlorobenzene solution of random copolymers and TTPTTT‐4F with a weight ratio of 1:1.2 and 0.25 vol% 1,8‐diiodooctane additive. The current density–voltage (*J*–*V*) curves are shown in **Figure** **3**a. It can be seen that upon decreasing the T1‐Cl contents in copolymers, the open‐circuit voltage (*V*_oc_) value of the resulting PSCs is gradually decreased from 0.90 to 0.82 V, which is mainly attributed to sequentially up‐shifted HOMO levels. Overall, the PT2‐based device yielded the highest PCE of 13.45%, with a *V*_oc_ of 0.88 V, a short‐circuit current (*J*_sc_) of 20.07 mA cm^−2^, and a fill factor (FF) of 76.4%. The calculated electron (*μ*_e_) and hole mobilities (*μ*_h_) of PT2:TTPTTT‐4F blend were measured to be 5.63 × 10^−4^ cm^2^ V^−1^ s^−1^, and 7.63 × 10^−4^ cm^2^ V^−1^ s^−1^, with a more balanced μ_h_/µ_e_ ratio of 1.36 than the other blends, which partially accounts for the high photovoltaic performance achieved in PT2:TTPTTT‐4F device. The external quantum efficiency (EQE) spectra are shown in Figure 3b. All the PSCs show broad photoresponse from 300 to 850 nm with the same absorption edge induced by TTPTTT‐4F acceptor. Among them, the PT2‐based device delivered a maximum EQE value of 78.90% at 680 nm. Further decreasing the T1‐Cl contents, the EQE value is gradually reduced. The calculated *J*_sc_ from the EQE spectra are summarized in **Table** **2**, which matches well with the values obtained from *J*–*V* measurement with a mismatch less than 5%. Moreover, we found that PT2 can work well with several representative NFAs, such as O‐INIC3,^[^ ^27^ ^]^ EH‐INIC3, and Y6^[^ ^28^ ^]^ (Figure S3, Supporting Information), and the corresponding PCEs of 14.14%, 13.47%, and 15.61% are achieved, respectively (Figure S5, Supporting Information, Table 2).

**Figure 3:**
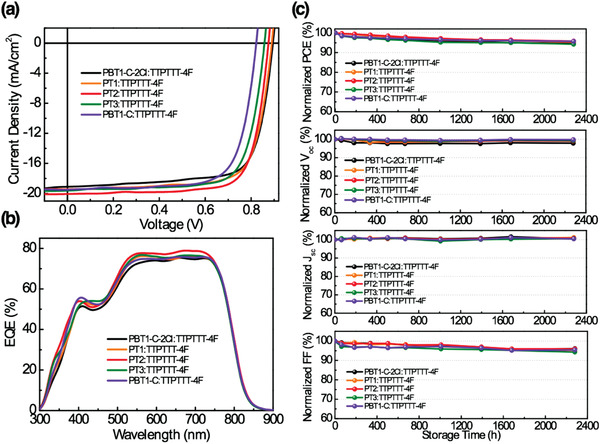
a) *J*–*V* characteristics and b) the corresponding EQE spectra of PSCs based on copolymers and TTPTTT‐4F. c) Degradation of normalized PCE, *V*_oc_, *J*_sc_, and FF of PSCs. The devices were unencapsulated and stored in air for more than 2000 h.

**Table 2:** Summary of device parameters of PSCs based on copolymers:TTPTTT‐4F, PT2:O‐INIC3, PT2:EH‐INIC3, and PT2:Y6 blends

Materials	*V*_oc_ [V]	*J*_sc_ [mA cm^−2^]	*J*_cal_ a ^)^ [mA cm^−2^]	FF [%]	PCE^b^^)^ [%]	PCE_max_ [%]
PBT1‐C‐2Cl:TTPTTT‐4F	0.90 ± 0.01	19.05 ± 0.23	18.29	73.5 ± 0.6	12.63 ± 0.11	12.77
PT1:TTPTTT‐4F	0.89 ± 0.01	19.22 ± 0.15	18.63	73.7 ± 1.0	12.84 ± 0.16	12.99
PT2:TTPTTT‐4F	0.88 ± 0.01	19.89 ± 0.20	19.21	75.7 ± 0.6	13.26 ± 0.19	13.45
PT3:TTPTTT‐4F	0.86 ± 0.01	19.42 ± 0.19	18.78	73.9 ± 0.5	12.45 ± 0.20	12.61
PBT1‐C:TTPTTT‐4F	0.82 ± 0.01	19.35 ± 0.16	18.70	72.1 ± 1.2	11.72 ± 0.07	11.82
PT2:O‐INIC3	0.93 ± 0.01	20.08 ± 0.12	19.31	74.6 ± 0.8	13.98 ± 0.15	14.14
PT2:EH‐INIC3	0.91 ± 0.01	20.12 ± 0.19	19.37	73.2 ± 0.6	13.33 ± 0.15	13.47
PT2:Y6	0.83 ± 0.01	25.77 ± 0.26	24.84	72.3 ± 0.7	15.53 ± 0.06	15.61

a) Integrated from EQE spectra;

b) The average PCE values were obtained from ten devices.

Notably, we measured the long‐term stability of the copolymers‐based PSCs. The unencapsulated devices were stored under ambient condition during the period of stability test. The normalized device parameters are provided in Figure 3c. All the TTPTTT‐4F‐based devices exhibited extraordinary ambient stability, maintaining ≈95% of initial PCE value after 2250 h (humidity ≈ 40%). The *V*_oc_ and *J*_sc_ keep nearly unchanged. The final PCE decrease is mainly caused by the slight decay of FF.

The aging AFM and GIWAXS measurements were performed to investigate the morphology stability. The relevant blends were first measured and then remeasured after storage in air after 2250 h. As presented in Figures S6 and S7, Supporting Information, it can be seen that the morphology, molecular orientation, and the degree of crystallization of all the blends remained nearly unchanged over time. All the blend films still exhibited clear fibrillar network structures, indicating excellent morphological stability of these blend films. We speculate that the well‐defined fibrillar structure may serve as medium to form stable frameworks similar to cross‐linked network,^[^ ^34^, ^35^ ^]^ which could inhibit the unfavorable aggregation and diffusion of molecules, affording excellent long‐term stability of the corresponding PSCs eventually. As shown in Figure S8, Supporting Information, all the PT2:O‐INIC3, PT2:EH‐INIC3, and PT2:Y6 blends also exhibit obvious fibrillar network structures. Resultingly, all the corresponding devices delivered good ambient stability (Figure S9, Supporting Information). After storage in air after 2350 h (humidity ≈ 40%), PT2:O‐INIC3, PT2:EH‐INIC3, and PT2:Y6 based device still remained 90.6%, 91.2%, and 87.3% of initial PCE values.

The photocurrent density (*J*_ph_) versus effective voltage (*V*_eff_) was conducted to investigate the exciton dissociation and charge collection properties of PSCs (Figure S10, Supporting Information). The charge dissociation probabilities *P*(*E*,*T*) were calculated to be 95.63%, 96.27%, 97.61%, 96.24%, and 96.08% for PBT1‐C‐2Cl, PT1, PT2, PT3, and PBT1‐C‐based PSCs, respectively, suggesting that the incorporation of T1‐Cl in copolymers could influence exciton dissociation ability.^[^ ^36^ ^]^ Moreover, the dependence of *J*_sc_ versus light intensity (*P*_light_) was measured. The power law between *J*_sc_ and *P*_light_ can be described by the formula of *J*_sc_ ∝ *P* ^*α*^, in which *α* is the exponential factor.^[^ ^37^ ^]^ The *α* values are 0.972, 0.994, 0.999, 0.987, and 0.978 for PBT1‐C‐2Cl, PT1, PT2, PT3, and PBT1‐C based PSCs, respectively, indicating negligible bimolecular recombination in PT1 and PT2 devices.

To clearly evaluate the changes of phase separation and fibril dimension of the blends, photoinduced force microscopy (PiFM) was carried out. PiFM has emerged as an effective technology to reveal the phase domains of BHJ films by imaging at the characteristic IR absorption wave numbers corresponding to the donor and acceptor materials.^[^ ^38^, ^39^, ^40^, ^41^ ^]^ Compared to the conventional AFM and TEM measurements, PiFM could clearly divide the phase region of the donor and the acceptor.^[^ ^42^ ^]^ The PiFM spectra of PBT1‐C‐2Cl, PT2, PBT1‐C, and TTPTTT‐4F neat films are shown in Figure S11, Supporting Information. Due to the similar chemical structure of copolymers, similar PiFM spectra were obtained. Regarding the PiFM measurements, we choose characteristic absorption peak at 1651 cm^−1^ for the copolymers and 1538 cm^−1^ for the TTPTTT‐4F, and then the copolymers and TTPTTT‐4F could be selectively imaged (Figure S12, Supporting Information). PiFM images of PBT1‐C‐2Cl:TTPTTT‐4F, PT2:TTPTTT‐4F, and PBT1‐C:TTPTTT‐4F blends are presented in **Figure** **4**. Distinct phase separation can be seen for all the blends. Prominent fibril networks arising from copolymers are evenly distributed in the whole blends and TTPTTT‐4F was inserted in‐between the fibril networks. This interpenetrating networks are favorable for charge transport.

**Figure 4:**
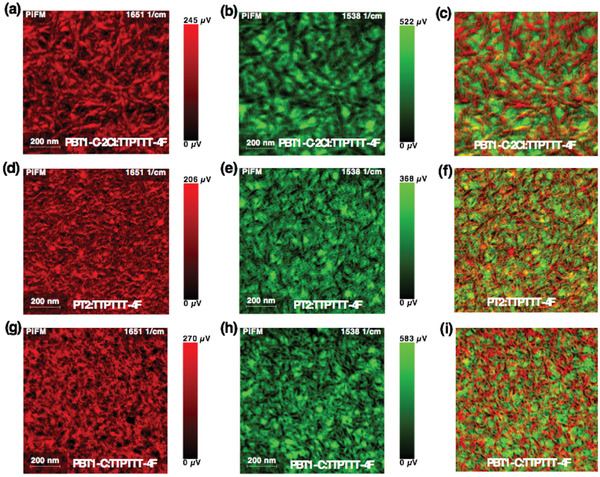
a–i) PiFM images of PBT1‐C‐2Cl:TTPTTT‐4F, PT2:TTPTTT‐4F, and PBT1‐C:TTPTTT‐4F blends based on IR absorption at different wave numbers, imaged at 1651 cm^−1^ for copolymer donors (a,d,g); imaged at 1538 cm^−1^ for the TTPTTT‐4F acceptor (b,e,h), and the combined images mapped at 1651 cm^−1^ and 1538 cm^−1^ (c,f,i).

To determine the fiber diameter, we performed image analysis on the PiFM images (Figures S13–S15, Supporting Information). For each PiFM image, we first filtered the image using a Gaussian filter to remove the excess noises. We then binarized the image into a black/white image. We used a threshold to ensure the area fractions of the foreground (donor) and background (acceptor) is equal. We carried out a medial axis transform to obtain the topological skeleton of the donor domains. The topological skeleton is one‐pixel wide skeleton representation of the donor domain with the same connectivity of the original object. After that we computed the shortest distance to the background for each point of the skeleton. Twice of the averaged distance gives the fiber diameter. As shown in Figures S13–S15, Supporting Information, the fiber diameter of PT2 is 18.0 nm, which is narrower than those of PBT1‐C‐2Cl (*d* = 24.0 nm) and PBT1‐C (*d* = 20.4 nm). Similarly, we can get the acceptor domain size. As a result, TTPTTT‐4F in PT2:TTPTTT‐4F blend shows domain size of 24.2 nm, relatively smaller than those in PBT1‐C‐2Cl:TTPTTT‐4F (*d* = 27.5 nm) and PBT1‐C: TTPTTT‐4F (*d* = 25.1 nm). The appropriate phase separation and smaller domain size in PT2:TTPTTT‐4F could facilitate efficient exciton dissociation and charge collection, therefore leading to improved *J*_sc_ and FF. The results testify that manipulating the T1‐Cl contents in copolymers can fine‐tune the molecular packing, crystallinity, and fibril diameter to achieve optimized BHJ morphology.

Transient absorption (TA) spectroscopy was employed to investigate the effect of crystallinity and molecular packing of the donor copolymers on the charge generation and dynamics in their blends with the TTPTTT‐4F acceptor.^[^ ^10^, ^43^ ^]^ The blend films of PBT1‐C‐2Cl, PT2, and PBT1‐C donors were excited at a pump wavelength of 550 nm where most of the photons were absorbed by the donors. **Figure** **5**a shows a series of transient absorption spectra of PT2:TTPTTT‐4F blend at different pump‐probe delay times at an excitation fluence of 3µJ cm^−2^. The spectra include both positive and negative signals representing ground state bleach (GSB) and photoinduced absorption (PIA) features, respectively. The spectra at early time scales represent the singlet excitons formed in the donor phase with a GSB around ≈2 eV and PIA at ≈1.2 eV as confirmed from the exciton spectra of the neat PT2 (Figure S16, Supporting Information). After ≈5 ps, a new red shifted PIA signature is originated around ≈1.3 eV, representing the free charge pairs, which persists beyond the 6 ns time window of this experiment. Similar spectral features are observed for the PBT1‐C‐2Cl and PBT1‐C blends (Figure S17, Supporting Information).

**Figure 5:**
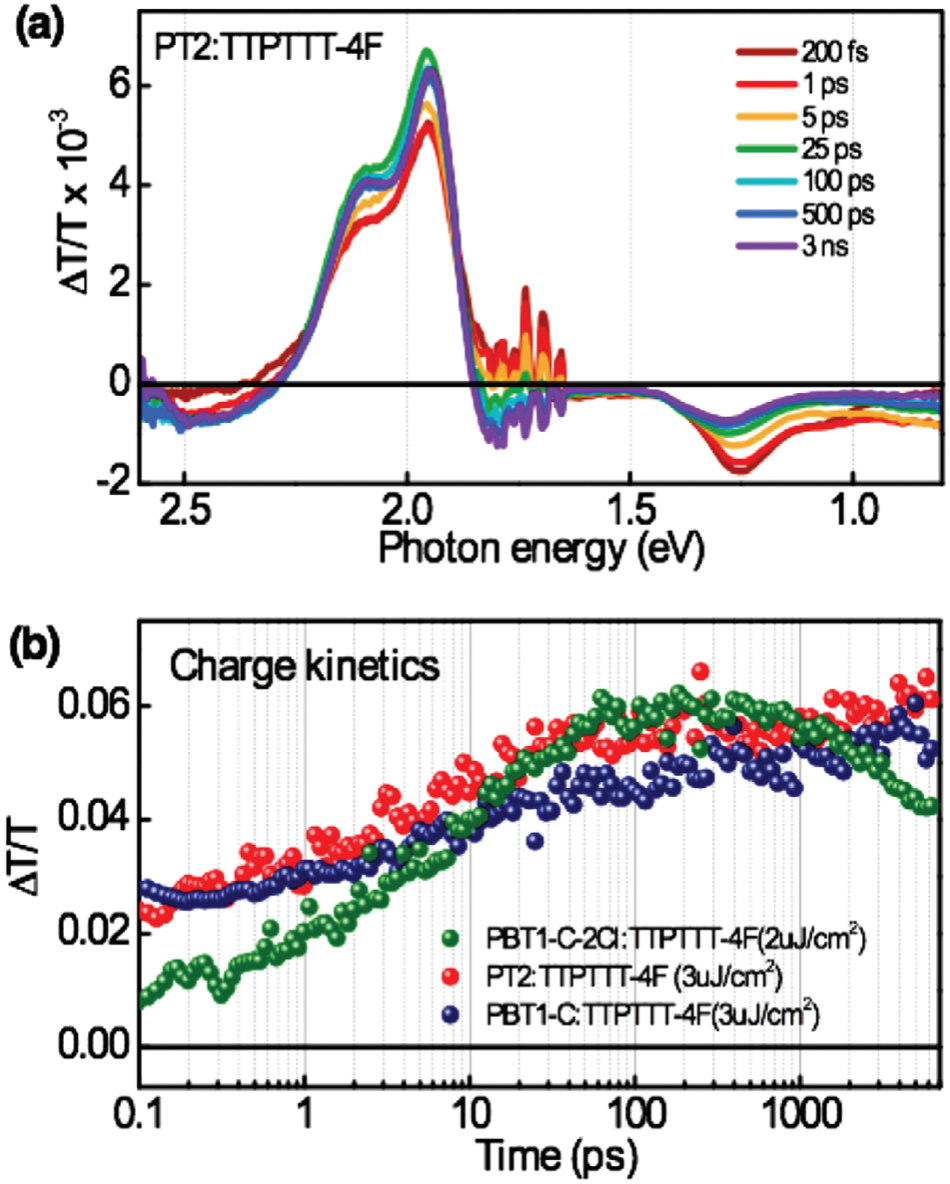
a) Transient absorption spectra of a PT2:TTPTTT‐4F blend, excited at 550 nm, at a pump fluence of 3 µJ cm^−2^. b) Kinetics of the charge‐based photoinduced absorption peak at 1.4 eV for PBT1‐C‐2Cl:TTPTTT‐4F, PT2:TTPTTT‐4F, and PBT1‐C: TTPTTT‐4F blend films (pump fluences are noted in brackets).

The charge generation dynamics in the three blends are compared in the Figure 5b. This plot is obtained via the bilinear decomposition of the blend TA surfaces by applying the neat exciton spectra of the donor materials as spectral masks. As per the plot, upon selectively exciting the donors, the charge growth is mostly associated with the exciton diffusion (see exciton kinetics Figure S18, Supporting Information) that peaks around 200 ps and then undergo recombination which can be explained by its higher crystallinity and thereby higher hole mobility that increases the probability of meeting of charge carriers in the donor phase. We also notice that some charge pairs are generated promptly which is consistent with the interpenetrating nanoscale network formed between the donor fibril networks and the acceptor molecules. The slow charge growth and the low fraction of prompt charges in the PBT1‐C‐2Cl:TTPTTT‐4F blend compared to the others is attributed to its larger fibril width and there by the large phase separation between the donor‐acceptor phases. Meanwhile, in the optimized PT2:TTPTTT‐4F blend, nearly half of the charge population is generated promptly, followed by a continuous growth without a recombination phase in the 6 ns time window we used, and is consistent with its appropriate phase separation, hole mobility, morphology, and thereby high efficiency.

In summary, a series of copolymers were designed and synthesized via a random copolymerization approach. The crystallinity, and morphological properties of copolymers can be effectively tuned by altering the T1‐Cl contents. When blended with TTPTT‐4F, PT2 was found to outperform the other copolymers with relatively higher photovoltaic performance due to its optimized fibril network morphology. Moreover, PT2 can work well with other high‐performance NFAs, such as O‐INIC3, EH‐INIC3, and Y6. The corresponding devices showed high PCEs of 14.14, 13.47, and 15.61%, respectively. The well‐defined fibril interpenetrating morphology with appropriate phase separation in PT2‐based blends can efficiently suppress the unfavorable aggregation, resulting in excellent morphological stability. As a result, PT2‐based PSCs possess superior ambient stability. Our work highlights the importance of morphology control in PSCs. Both high efficiency and long‐term stability of PSCs can be realized by optimizing the fibril network morphology of the active layer.

## Experimental Section

##### Materials

PBT1‐C, PBT1‐C‐2Cl, TTPTTT‐4F, O‐INIC3, and PT2 were synthesized in the lab according to the literature.^[^ ^10^, ^24^, ^27^, ^28^, ^44^, ^45^ ^]^ It should be noted that both O‐INIC3 and EH‐INIC3 shared the same central core with originally reported INIC3,^[^ ^44^ ^]^ but differed in side chains. And the coplymers PT1 and PT3 were synthesized by changing the T1‐2Cl/T1 ratios according to the literature.^[^ ^45^ ^]^ Y6 was purchased from HYPER Inc. (Zhejiang, China). Zinc acetate dihydrate (Zn(CH_3_COO)_2_·2H_2_O) was purchased from Alfa Aesar. Chlorobenzene, chloroform, 1,8‐diiodooctane (DIO), ethanolamine (NH_2_CH_2_CH_2_OH), and 2‐methoxyethanol (CH_3_OCH_2_CH_2_OH) were purchased from Sigma‐Aldrich. Other reagents and solvents were purchased from commercial sources and were used without further purification unless stated otherwise.

##### Solar Cell Fabrication and Characterization

PSCs were fabricated with inverted device architecture of ITO/ZnO/photoactive layer/MoO_3_/Ag. The ITO‐coated glass substrates were sequentially cleaned by ultrasonic treatment in detergent deionized water, acetone, and isopropyl alcohol for 20 min, respectively. After drying for one night, a ZnO layer was generated by spin coating the ZnO precursors solution on the top of ITO at 4000 rpm for 30 s and then baked at 200 °C for 15 min under ambient conditions. ZnO precursor was prepared by dissolving 1 g zinc acetate dihydrate and 280 µL ethanolamine in 10 mL 2‐methoxyethanol under stirring for 12 h for the hydrolysis reaction. The mixed solutions of copolymers and NFAs such as TTTPTT‐4F, O‐INIC3, or EH‐INIC3 were prepared by dissolving polymer donors and NFAs in CB with a weight ratio of 1:1.2, and 0.25 vol% 1,8‐diiodooctane additive. The mixed solution of PT2 and Y6 were prepared by dissolving PT2 and Y6 in CF with a weight ratio of 1:1.2, and 0.25 vol% 1,8‐diiodooctane additive. The prepared solutions were stirred overnight prior to cast. The active layers with an optimal thickness of 110 nm were generated by spin coating the mixed solutions of donor and acceptor, followed by thermal annealing at 100 °C for 10 min in a N2‐ filled glovebox. Finally, 7 nm MoO_3_ and 90 nm Ag layer was thermally deposited under the vacuum condition of 2 × 10^−4 ^Pa. The active area of PSCs is 4.00 mm^2^. Current density–voltage (*J*–*V*) characteristics were measured using a Keithley 2400 Source Measure Unit. The currents were measured under 100 mW cm^−2^ simulated 1.5 Global (AM 1.5 G) solar simulator (Enli Technology Co., Ltd, SS‐F5‐3A). The light intensity was calibrated by a standard Si solar cell (SRC‐ 2020, Enli Technology Co., Ltd). EQE spectra were performed on a solar‐cell spectral‐response measurement system (QE‐R, Enlitech).

## Conflict of Interest

The authors declare no conflict of interest.

## Supporting information

Supporting Information
