# Supplementary material for: An Optimized Fibril Network Morphology Enables High‐Efficiency and Ambient‐Stable Polymer Solar Cells
Source: Adv Sci (Weinh). 2020 Jul 26;7(18):2001986. doi: 10.1002/advs.202001986 (PMC7509652; doi:10.1002/advs.202001986)
Supplement: Supplementary file 1 — Supporting Information [file ADVS-7-2001986-s001.pdf]

Copyright WILEY-VCH Verlag GmbH & Co. KGaA, 69469 Weinheim, Germany, 2020.

ADVANCED SCIENCE
Open Access

Supporting Information

for *Adv. Sci.,* DOI: 10.1002/advs.202001986

An Optimized Fibril Network Morphology Enables High-Efficiency and Ambient-Stable Polymer Solar cells

*Jiali Song, Linglong Ye, Chao Li, Jinqiu Xu, Sreelakshmi Chandrabose, Kangkang Weng, Yunhao Cai, Yuanpeng Xie, Padraic O'Reilly, Kai Chen, Jiajia Zhou, Yi Zhou, Justin M. Hodgkiss, Feng Liu, and Yanming Sun**

Supporting Information

An Optimized Fibril Network Morphology Enables High-Efficiency and Ambient-Stable Polymer Solar cells

*Jiali Song, Linglong Ye, Chao Li, Jinqiu Xu, Sreelakshmi Chandrabose, Kangkang Weng, Yunhao Cai, Yuanpeng Xie, Padraic O'Reilly, Kai Chen, Jiajia Zhou, Yi Zhou, Justin M. Hodgkiss, Feng Liu, and Yanming Sun**

J. Song, Dr. L. Ye, Dr. C. Li, Dr. Y. Cai, Y. Xie, Prof. J. Zhou, Prof. Y. Sun

School of Chemistry

Beihang University

Beijing 100191, P. R. China

Email: sunym@buaa.edu.cn

J. Xu, Prof. F. Liu

Department of Polymer Science and Engineering

School of Chemistry and Chemical Engineering

Shanghai Jiao Tong University

Shanghai 200240, P. R. China

S. Chandrabose, Dr. K. Chen, Prof. J. Hodgkiss

MacDiarmid Institute for Advanced Materials and Nanotechnology

and School of Chemical and Physical Sciences

Victoria University of Wellington

Wellington 6010, New Zealand

Prof. Y. Zhou
Laboratory of Advanced Optoelectronic Materials
College of Chemistry
Chemical Engineering and Materials Science
Soochow University,
Suzhou 215123 , P. R. China

P. O'Reilly
Molecular Vista Inc.
6840 Via Del Oro
Suite 110
San Jose, CA 95119

Random Copolymerization of Copolymers

PBT1-C, PBT1-C-2Cl, and PT2 were synthesized according to the literature.[1-3] And the coplymers PT1 and PT3 were synthesized by changing the T1-2Cl/T1 ratios according to the literature.[3] The molecular weight (M_n) and the polydispersity index (PDI) , *etc.* of PBT1-C-2Cl, PT1, PT2, PT3, and PBT1-C used in this study are as follows:

PBT1-C-2Cl: M_n = 20.8 kDa, PDI = 2.53. Elemental analysis: $C_{80}H_{96}Cl_2O_2S_6$: calcd. C 71.02, H 7.15, Cl 5.24; found: C 69.80, H 7.09, Cl 5.41.

PT1: M_n = 30.2 kDa, PDI = 2.30. Elemental analysis: $C_{80}H_{96.2}Cl_{1.8}O_2S_6$: calcd. C 71.39, H 7.20, Cl 4.74; found: C 70.11, H 7.19, Cl 4.82.

PT2: M_n = 41.1 kDa, PDI = 2.38. Elemental analysis: $C_{80}H_{96.6}Cl_{1.4}O_2S_6$: calcd. C 72.13, H 7.31, Cl 3.73; found: C 71.36, H 7.30, Cl 3.98.

PT3: M_n = 51.2 kDa, PDI = 2.50. Elemental analysis: $C_{80}H_{97}ClO_2S_6$: calcd. C 72.88, H 7.42, Cl 2.69; found: C 72.74, H 7.48, Cl 2.84.

PBT1-C: M_n = 68.9 kDa, PDI = 2.86. Elemental analysis: $C_{80}H_{98}O_2S_6$: calcd. C 74.83, H 7.69; found: C 74.67, H 7.76.

Synthesis of EH-INIC3

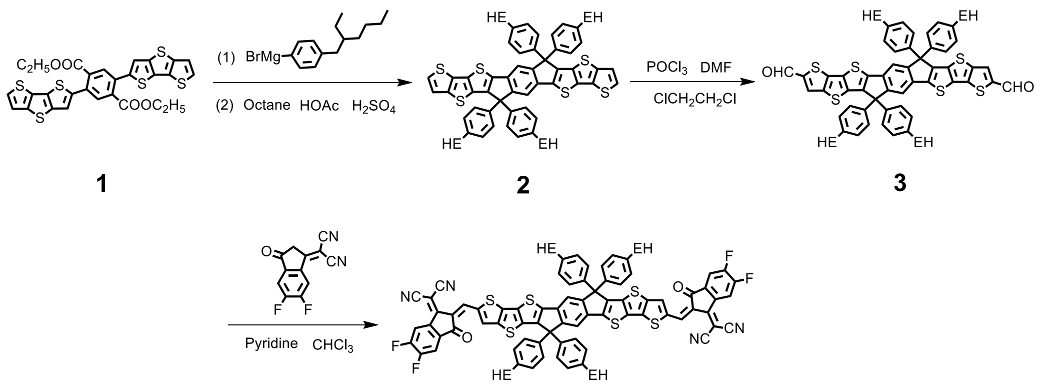

EH-INIC3

Scheme S1. Synthesis routes of EH-INIC3, which are similar to our reported literature.[4]

Compound 2: ^{1}H NMR (300 MHz, $CDCl_3$, δ): 7.53 (s, 2H), 7.31-7.29 (d, 2H), 7.22-7.20 (t, 10H), 7.08-7.05 (d, 8H), 2.50-2.47 (d, 8H), 1.55 (m, 4H), 1.24-1.23 (m, 24H), 0.87-0.82 (t, 24H); ^{13}C NMR (100 MHz, $CDCl_3$, δ): 153.51, 147.51, 141.85, 141.39, 140.82, 139.97, 136.20, 135.97, 132.57, 131.67, 129.32, 127.99, 125.48, 120.70, 116.76, 63.16, 40.83, 39.75, 32.36, 29.74, 28.86, 25.50, 23.01, 14.13, 10.77; HRMS (MALDI-TOF) *m*/*z*: $[M + H]^+$ calcd for $C_{80}H_{90}S_6$, 1242.54; found, 1243.2.

Compound 3: ^{1}H NMR (300 MHz, $CDCl_3$, δ): 9.90 (s, 2H), 7.83 (s, 2H), 7.60 (s, 2H), 7.20-7.18 (d, 8H), 7.10-7.08 (d, 8H), 2.51-2.49 (d, 8H), 1.57-1.55 (m, 4H), 1.25-1.23 (m, 24H), 0.87-0.83 (t, 24H). ^{13}C NMR (100 MHz, $CDCl_3$, δ): 182.58, 154.27, 147.49, 145.70, 143.21, 141.27, 141.12, 140.68, 139.34, 138.67, 136.25, 132.67, 130.05,

129.53, 127.86, 117.51, 63.30, 40.87, 39.75, 32.37, 29.74, 28.86, 25.54, 23.00, 22.74, 14.13, 10.79; HRMS (MALDI-TOF) *m*/*z*: $[M + H]^+$ calcd for $C_{82}H_{90}O_2S_6$, 1298.53; found, 1299.2.

EH-INIC3: ^1H NMR (300 MHz, $CDCl_3$, δ): 8.91 (s, 2H), 8.55-8.51 (m, 2H), 7.98(s, 2H), 7.70-7.66 (t, 4H), 7.20-7.18 (d, 8H), 7.12-7.10 (d, 8H), 2.52-2.50 (d, 8H), 1.55 (m, 4H), 1.24 (m, 24H), 0.87-0.84 (t, 24H); ^{13}C NMR (100 MHz, $CDCl_3$, δ): 185.05, 156.94, 155.21, 155.06, 154.02, 151.80, 151.63, 147.97, 147.40, 145.63, 142.58, 141.95, 140.46, 137.81, 137.18, 136.80, 135.64, 133.37, 133.32, 132.12, 128.59, 126.72, 120.18, 116.96, 114.04, 113.75, 113.16, 111.70, 111.45, 68.74, 62.26, 39.81, 38.67, 31.28, 28.68, 27.78, 24.44, 21.94, 13.08, 9.72; HRMS (MALDI-TOF) *m*/*z*: $[M + H]^+$ calcd for $C_{106}H_{94}F_4N_4O_2S_6$, 1722.56; found, 1723.8.

Material and Device Characterization

Nuclear magnetic resonance (NMR) spectra were recorded on a Bruker Avance 300 spectrometer using deuterated chloroform ($CDCl_3$) as the solvent and trimethylsilane (TMS) as the internal reference at room temperature. UV-vis absorption spectra were measured using a UV-vis spectrophotometer (Shimadzu UV-2700). Cyclic voltammetry (CV) measurements were conducted under nitrogen atmosphere at a scan rate of 100 mV s^{-1} using a Zahner IM6e Electrochemical workstation. A platinum plate coated with sample film was used as a working electrode, a platinum wire was used as a counter electrode, a saturated Ag/AgCl electrode was used as a reference electrode, 0.1 M tetra-n-butylammonium hexafluorophosphate (Bu_4NPF_6) in anhydrous acetonitrile solution was employed as a supporting electrolyte and Ferrocene/ferrocenium (Fc/Fc^+) is used as an internal standard. The onset oxidation potential of ferrocene external standard was determined to be 0.43 eV in our lab. Therefore, the HOMO and LUMO energy level could be calculated from the following equations: HOMO = – (E_{ox}^{onset} + 4.37) eV and LUMO = – (E_{red}^{onset} + 4.37) eV, where E_{ox}^{onset} and E_{red}^{onset} were the onset oxidation

potential and onset reduction potential relative to Ag/AgCl, respectively. Atomic force microscopy (AFM) measurements were carried out using a Dimension Icon AFM (Bruker) in the tapping mode. The charge transport properties were evaluated via space charge limited current (SCLC) method. The electron-only devices were fabricated with a structure of ITO/ZnO/photoactive layer/ZrAcac/Al, and the hole-only devices are fabricated with a structure of ITO/PEDOT:PSS/photoactive layer/Ag. The J–V curves of devices were fitted by using the Mott–Gurney equation: $J = 9\varepsilon_0\varepsilon_r\mu V^2/8L^3$, where J is the current density, ε_0 is the permittivity of free space, ε_r is the permittivity of the active layer, μ is the hole mobility or electron mobility, V is the internal voltage of the device ($V = V_{app} - V_{bi}$), where V_{app} is the applied voltage, V_{bi} is the offset voltage (V_{bi} is 0 V here), and L is the film thickness of the active layer. The eletron mobility or hole mobility could be calculated from the slope of the $J^{0.5}$–V curve. GIWAXS measurements were performed at beamline 7.3.3 at the Advanced Light Source, Lawrence Berkeley National Laboratory.

Photo-induced Force Microscopy (PiFM)

The PiFM images and spectra were carried out by Molecular Vista Inc., in partnership with Anfatec Instruments AG (Oelsnitz, Germany). PiFM is a multimodal Atomic Force Microscopy (AFM) technique, combining the high resolution of AFM with IR spectroscopy to obtain topographical and molecular information with sub-10 nm resolution.[5] A quantum cascade laser (QCL) is focused at the interface between the sample and a metallic-coated AFM tip in dynamic non-contact mode, with an average laser power of 100s μW on the sample surface and elliptical spot size from the off-axis parabolic mirror of $\lambda \times 1.5\lambda$. The QCL is tunable over the molecular fingerprint region, 770 to 1885 cm^{-1}. Upon IR absorption by the sample, attraction between the photoinduced dipole in the sample and the mirrored dipole in the metallic tip, and modulation of the van der Waals force gradient during thermal expansion of the sample, both result in a photo-induced force (PiF) that is measured by the

instrument.[6]

All measurements were generated using a VistaScope microscope from Molecular Vista Inc., coupled with a LaserTune QCL from Block Engineering, with a tunable range of 775 to 1885 cm^{-1} and spectral line width of 2 cm^{-1}. Gold-coated NCH 300 kHz non-contact cantilevers from Nanosensors were also used for all measurements. The PiFM images (Figure 4) were generated at a resolution of 256x256 pixels, line speed of 0.89 line/s and a size of 1 μm^2, at wavenumbers of 1651 cm^{-1} and 1538 cm^{-1} to highlight the copolymer donor and acceptor, respectively. The PiFM spectra in Figure S11 for TTPTTT-4F, PBT1-C-Cl, PT2, PBT1-C and ZnO were each the average of 6 point spectra on the sample surface, with an acquisition time of 30 s per point spectrum over a range of 800 to 1885 cm^{-1}. The PiFM spectra in Figure S12 for PT2 and PBT1-C blends were each acquired with a sweep time of 20 s per spectrum over a range of 1280 to 1885 cm^{-1}. The spectra for the PBT1-C-2Cl blend were acquired with a sweep time of 30 s per spectrum over a range of 800 to 1885 cm^{-1}, although only 1280 to 1885 cm^{-1} is displayed in Figure 12.

Transient absorption spectroscopy

Femtosecond transient absorption measurements are performed using the output of an amplified Ti: sapphire laser (Spitfire Spectra Physics) emitting pulses centred at 800 nm with 100 fs time duration and at a repetition rate of 3 KHz. The pump pulses at 550 nm are generated using an optical parametric amplifier (TOPAS) with the 800 nm fundamental input and then chopped at 1.5 KHz using a mechanical chopper to produce pump on and off pulses. The photoexcitations are probed via a broadband white light continuum generated by focusing a portion of the 800 nm fundamental to a 2 mm thick undoped YAG (Yttrium Aluminium Garnet) crystal. The polarizations of the pump and probe pulses are set at the magic angle (54.7°) to avoid the orientational effects. After passing through the photoexcited sample, the probe pulses are dispersed using a prism spectrometer and then collected by a visible CMOS line scan camera

and an InGaAs photodiode array (IR components). The time delay between the pump and probe pulses is obtained via a retroreflector connected a motorized translational stage. The differential transmission signals at various pump-probe delay times are calculated using sequential probe shots corresponding to pump on versus off. For all the measurements, 6000 shots were averaged at each time delays and is repeated for at least 3 times. The data saved as binary files are processed using MATLAB software including chirp and background corrections. All the samples were measured under continues vacuum to avoid degradation.

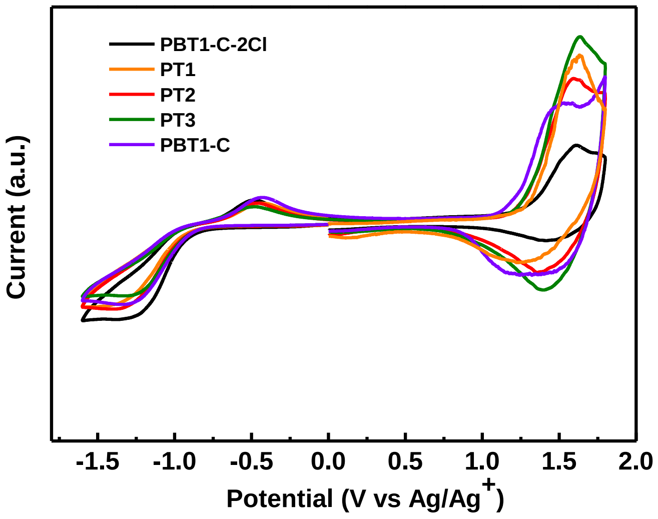

Figure S1. The electrochemical cyclic voltammograms of PBT1-C-2Cl, PT1, PT2, PT3, and PBT1-C.

Table S1. Optical and electrochemical properties of PBT1-C-2Cl, PT1, PT2, PT3, and

PBT1- C.

Materials	λ_{max}^{a} [nm]	λ_{max}^{b} [nm]	λ_{onset}^{b} [nm]	E_g^{c} [eV]	E_{ox} [V]	HOMO [eV]	E_{red} [V]	LUMO [eV]
PBT1-C-2Cl	628	632	684	1.81	1.17	-5.59	-0.90	-3.52
PT1	625	629	682	1.82	1.15	-5.57	-0.92	-3.50
PT2	618	625	680	1.82	1.13	-5.55	-0.91	-3.51
PT3	612	622	677	1.83	1.10	-5.52	-0.91	-3.51
PBT1-C	602	610	676	1.83	1.06	-5.48	-0.91	-3.51

[a]In chlorobenzene solution; b) in thin film; c) estimated from empirical formula: $E_g^{opt} = 1240/\lambda_{onset}$.

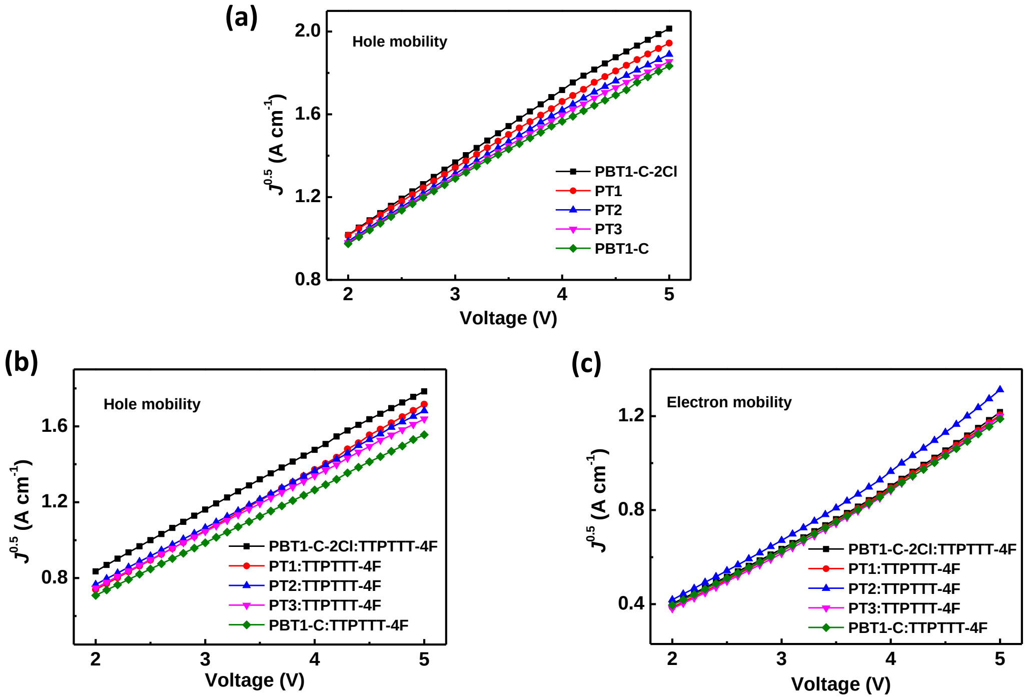

Figure S2. $J^{0.5}$-V curves of a) hole-only device based on PBT1-C-2Cl, PT1, PT2, PT3, and PBT1-C neat films; b) hole-only device of corresponding blends; c) electron-only

device of corresponding blends.

Table S2. Charge transpot properties of PBT1-C-2Cl, PT1-PT3, PBT1-C and their corresponding blends.

Materials	μ_e [cm^2 V^{-1} s^{-1}]	μ_h [cm^2 V^{-1} s^{-1}]	μ_h/μ_e
PBT1-C-2Cl		1.12×10^{-3}	
PT1		1.00×10^{-3}	
PT2		9.39×10^{-4}	
PT3		8.77×10^{-4}	
PBT1-C		8.14×10^{-4}	
PBT1-C-2Cl:TTPTTT-4F	5.27×10^{-4}	8.62×10^{-4}	1.63
PT1:TTPTTT-4F	5.32×10^{-4}	8.56×10^{-4}	1.60
PT2:TTPTTT-4F	5.63×10^{-4}	7.63×10^{-4}	1.36
PT3:TTPTTT-4F	5.32×10^{-4}	7.42×10^{-4}	1.39
PBT1-C:TTPTTT-4F	5.12×10^{-4}	7.15×10^{-4}	1.40

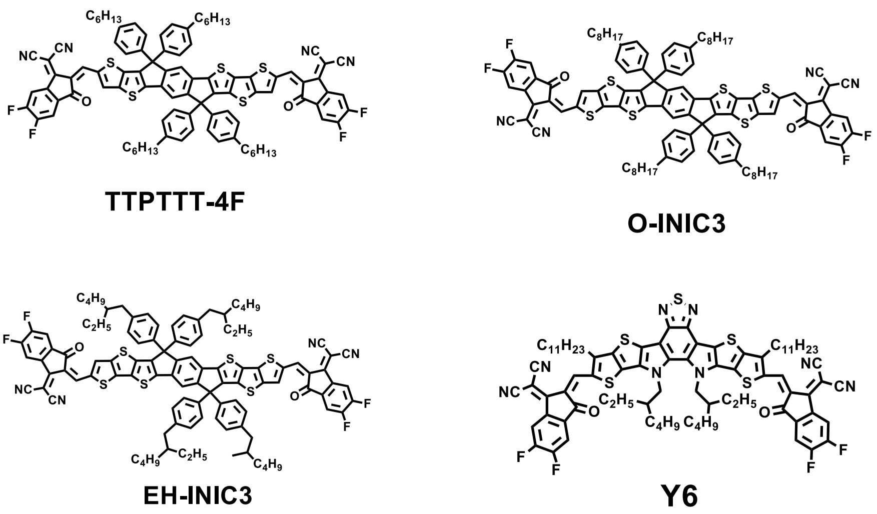

Figure S3. Chemical structure of TTPTTT-4F, O-INIC3, EH-INIC3 and Y6.

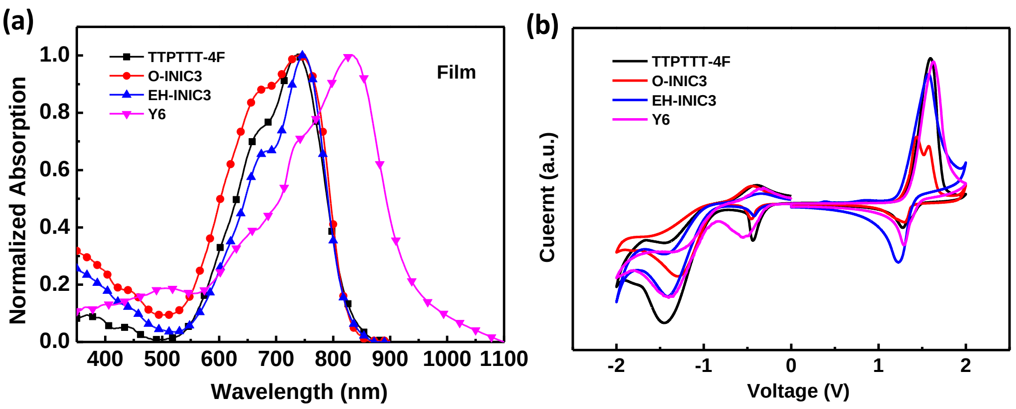

Figure S4. a) Normalized absorption and b) electrochemical cyclic voltammogram of

TTPTTT- 4F, O-INIC3, EH-INIC3 and Y6.

Table S3. Optical and electrochemical properties of TTPTTT-4F, O-INIC3, EH-INIC3 and Y6 NFAs.

Materials	λ_{max}[a] [nm]	λ_{max}[b] [nm]	E_g[c] [eV]	E_{ox} [V]	HOMO [eV]	E_{red} [V]	LUMO [eV]
TTPTT-4F	739	820	1.52	1.30	-5.68	-0.29	-4.09
O-INIC3	741	824	1.50	1.27	-5.65	-0.38	-4.00
EH-INIC3	741	822	1.51	1.30	-5.68	-0.34	-4.04
Y6	831	938	1.32	1.32	-5.70	-0.32	-4.06

[a]In chlorobenzene solution; [b]In thin film; [c]Estimated from empirical formula: $E_g^{opt} = 1240/\lambda_{onset}$.

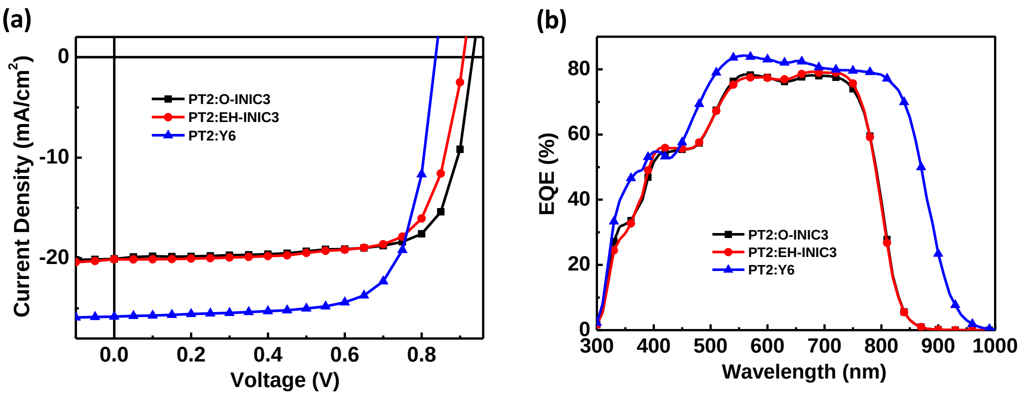

Figure S5. a) *J–V* characteristics and b) the corresponding EQE spectra of

PT2:O-INIC3, PT2:EH-INIC3 and PT2:Y6 based devices.

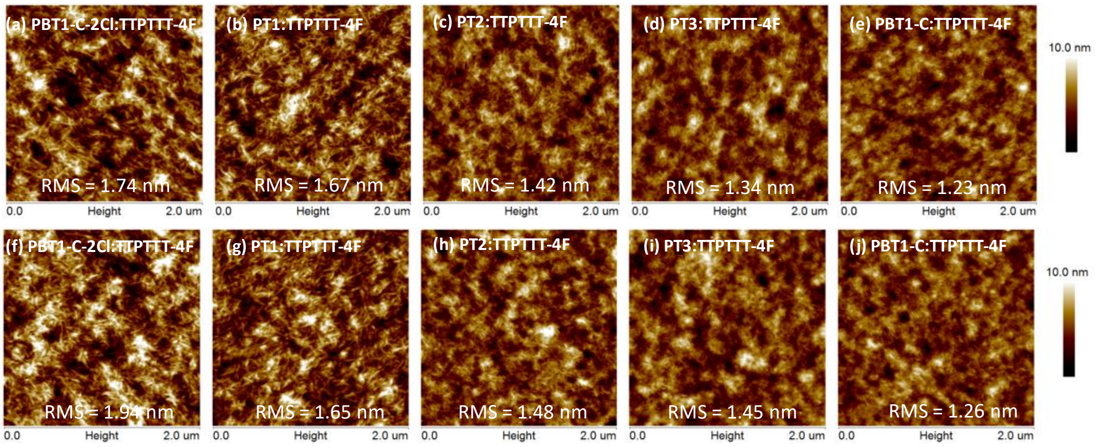

Figure S6. The AFM height and phase images (2 μm × 2 μm) of PBT1-C-2Cl, PT1, PT2, PT3, and PBT1-C blends a-e) freshly prepared and f-j) after 2250h in air conditions.

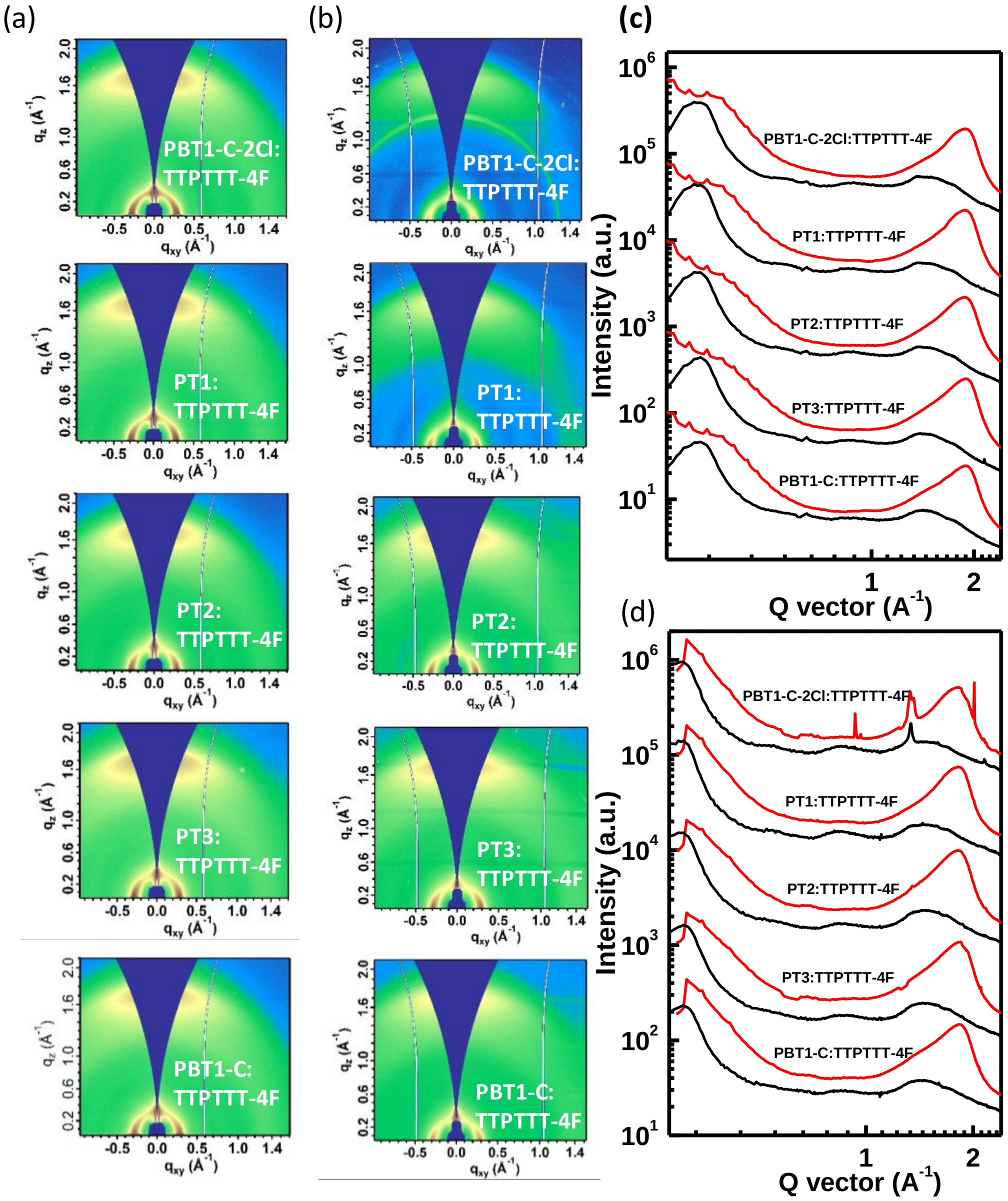

Figure S7. 2D GIWAXS pattern of a) PBT1-C-2Cl, PT1, PT2, PT3 and PBT1-C and c) their corresponding out-of-plane (red line) and in-plane (black line) line-cut profiles. Aging 2D GIWAXS pattern of b) and d) their corresponding out-of-plane (red line) and in-plane (black line) line-cut profiles. The same samples were measured after 2250 h in air for comparison.

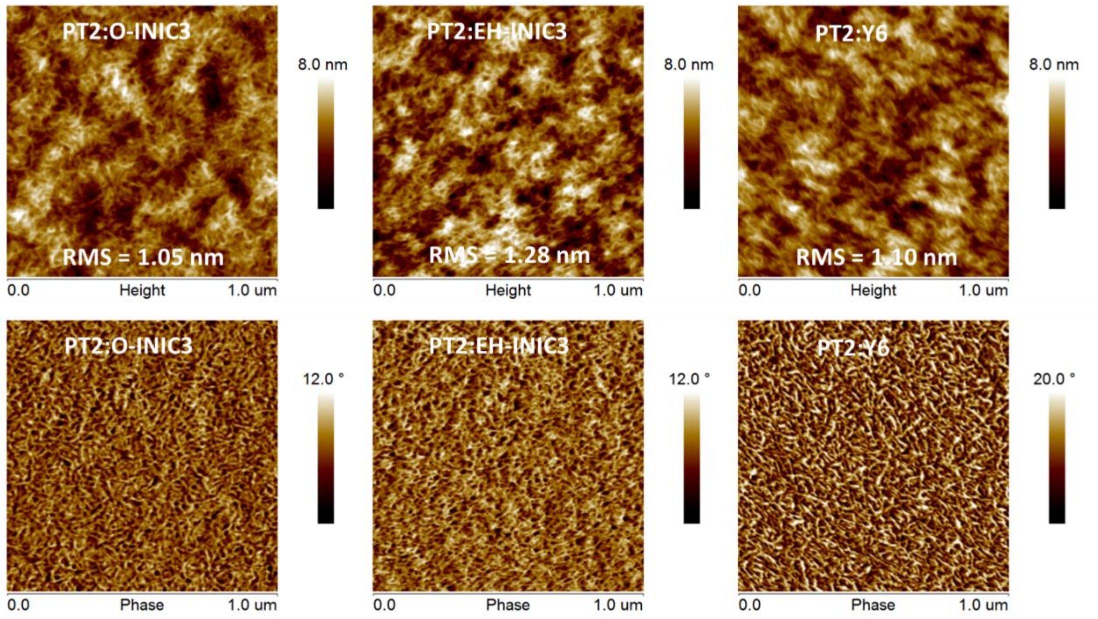

Figure S8. The AFM height and phase images of PT2:O-INIC3, PT2:EH-INIC3 and PT2:Y6 blend films.

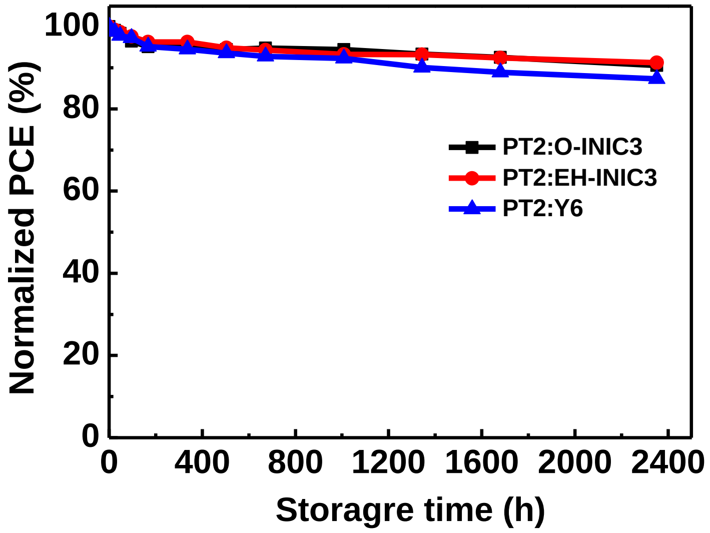

Figure S9. Degradation of normalized PCE of PT2:O-INIC3, PT2:EH-INIC3 and

PT2:Y6 based devices. The devices were encapsulated and stored in air.

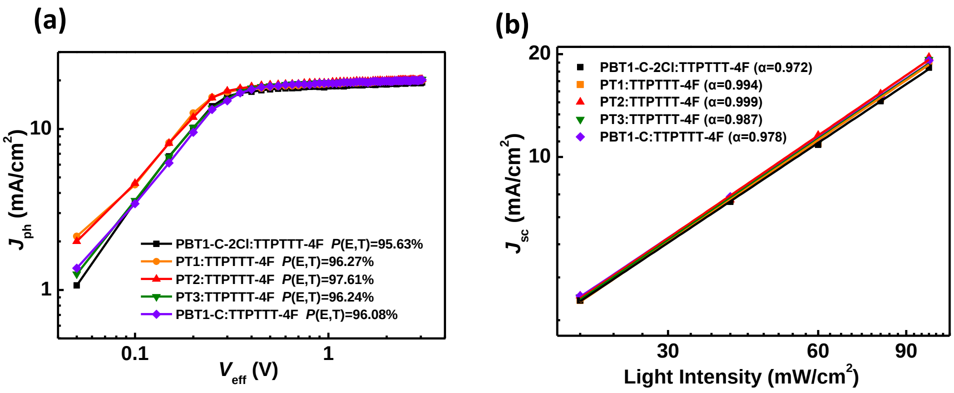

Figure S10. a) J_{ph} versus V_{eff} plot and b) light intensity dependence of J_{sc} of PBT1-C-2Cl, PT1, PT2, PT3 and PBT1-C based devices.

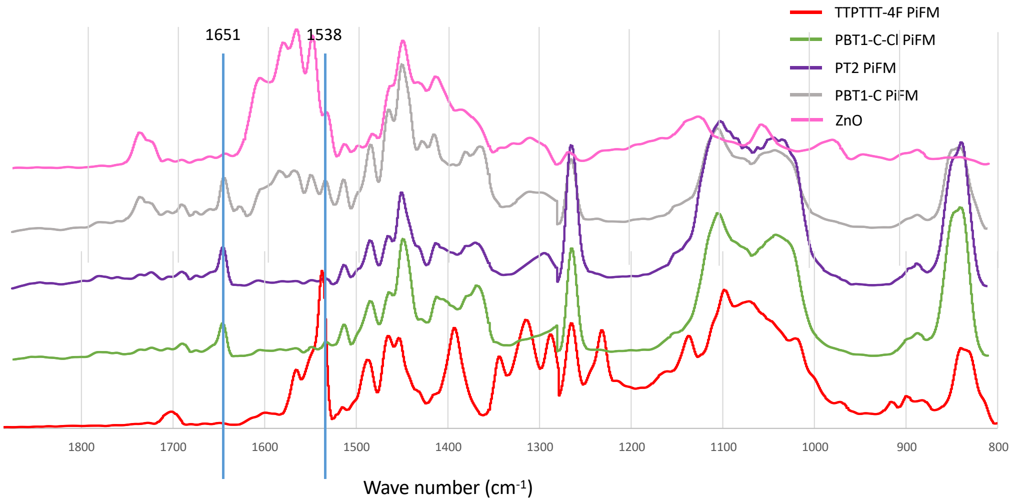

Figure S11. There is absorption at 1651 cm^{-1} for copolymers that is not found in the TTPTTT-4F sample. 1538 cm^{-1} is prominent on the TTPTTT-4F. Neither of these peaks are prominent on ZnO. These wavenumbers were used to highlight copolymers (1651 cm^{-1}) and TTPTTT-4F (1538 cm^{-1}) in the blends.

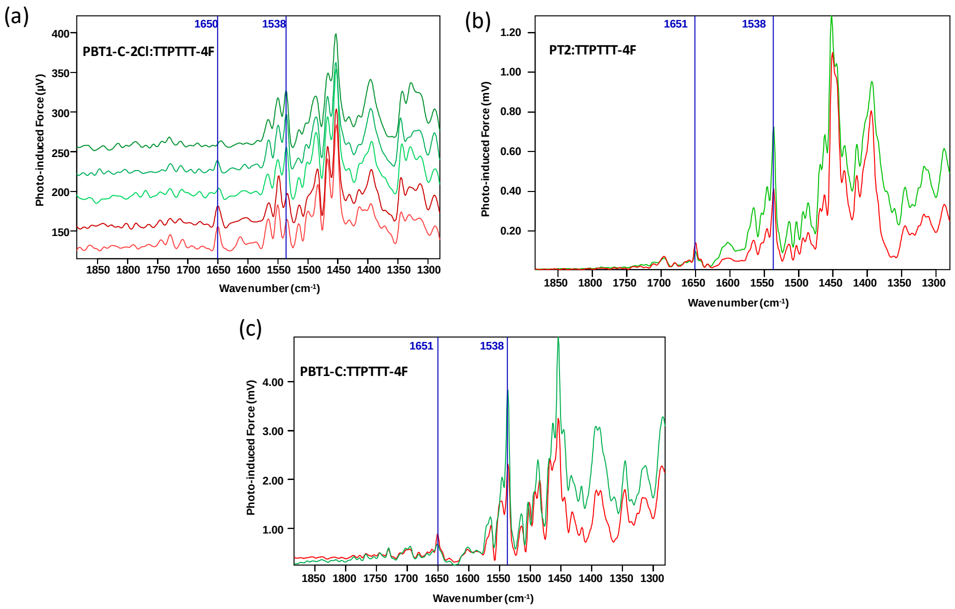

Figure S12. PiFM spectra of a) PBT1-C-2Cl, b) PT2 and c) PBT1-C blends.

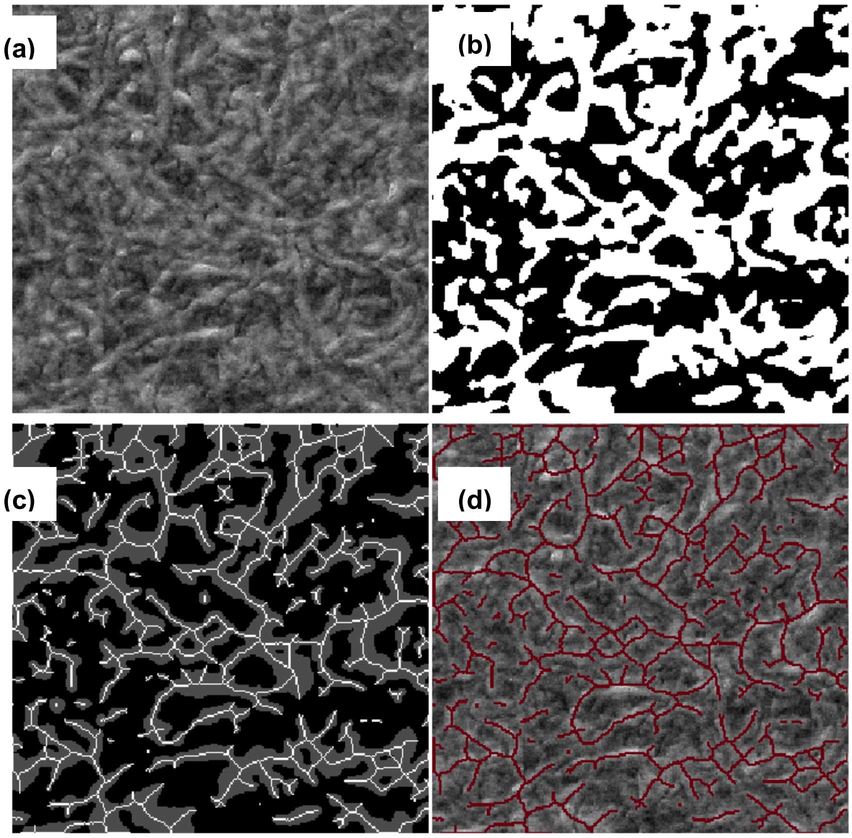

Figure S13. Step-by-step image analysis procedure on PBT1-C-2Cl/TTPTTT-4F sample. a) Raw PiFM image, where light regions indicate the polymer donor; b) binarized black/white image; c) topological skeleton; (d) skeleton overlaid with raw PiFM image.

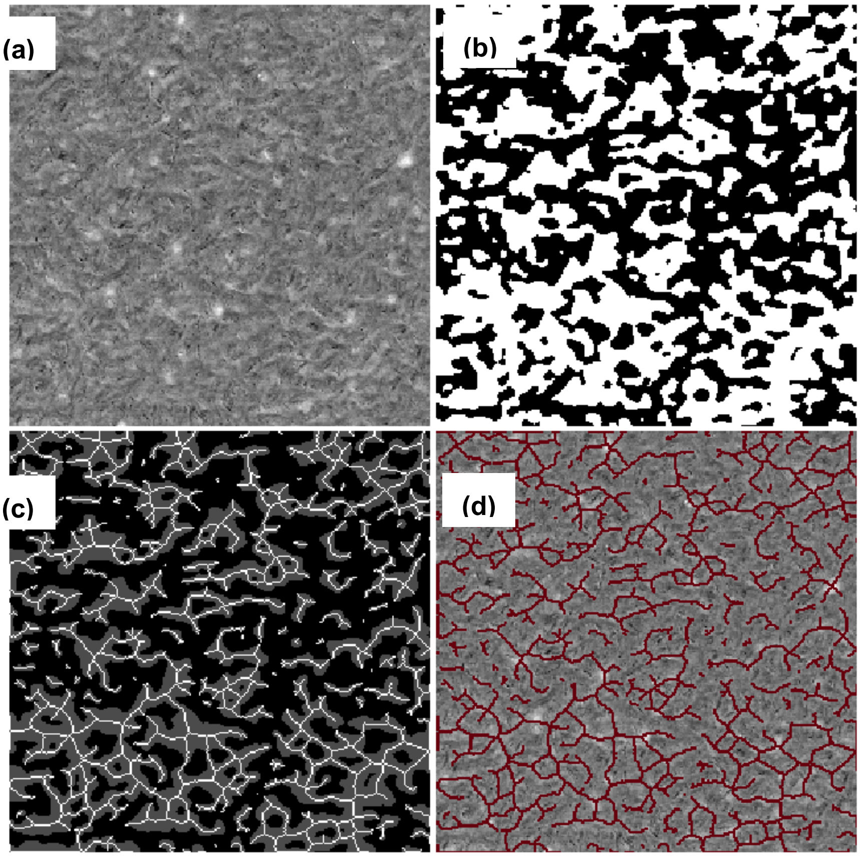

Figure S14. Step-by-step image analysis procedure on PT2/TTPTTT-4F sample. a) Raw PiFM image, where light regions indicate the polymer donor; b) binarized black/white image; c) topological skeleton; d) skeleton overlaid with raw PiFM image.

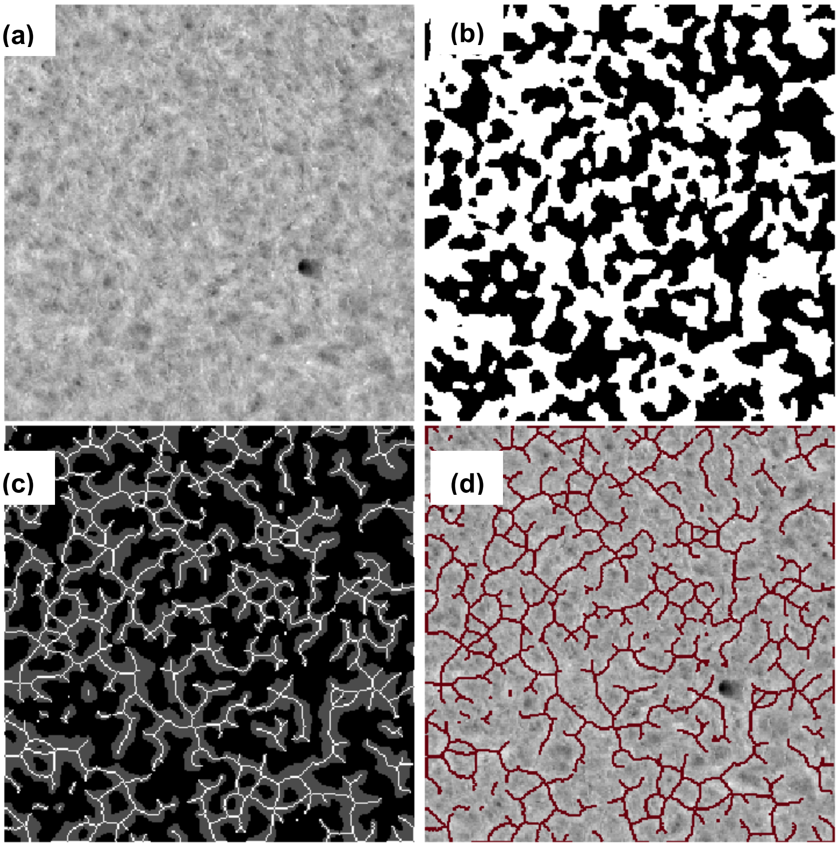

Figure S15. Step-by-step image analysis procedure on PBT1-C/TTPTTT-4F sample. a) Raw PiFM image, where light regions indicate the polymer donor; b) binarized black/white image; c) topological skeleton; d) skeleton overlaid with raw PiFM image.

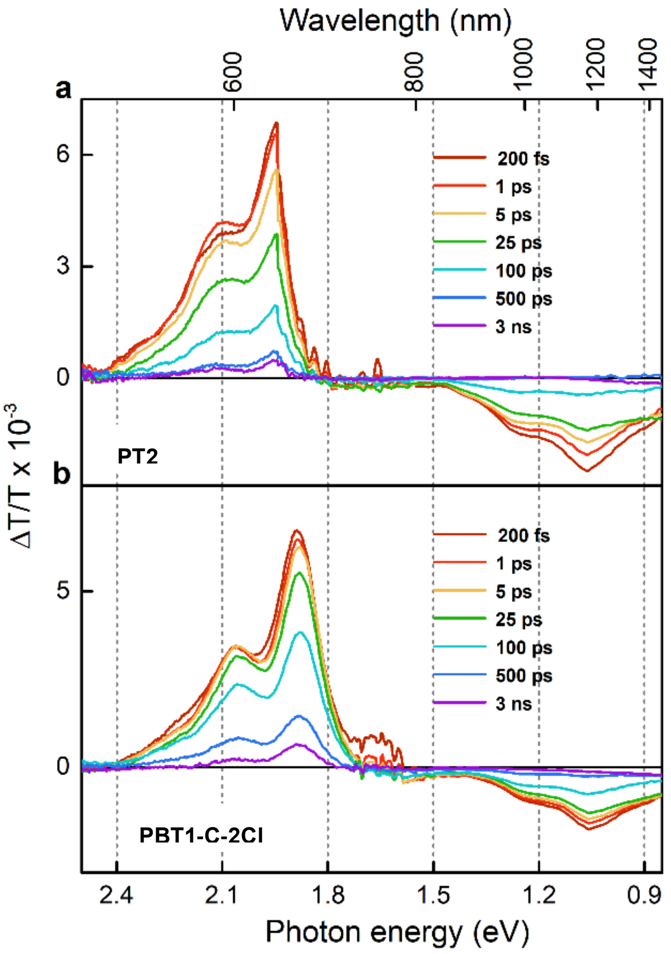

Figure S16. Series of transient absorption spectra of a) PT2 and b) PBT_1-C-2Cl neat films following excitation at 550 nm, at pump fluences of 3 μJ/cm^2 and 2 μJ/cm^2 respectively.

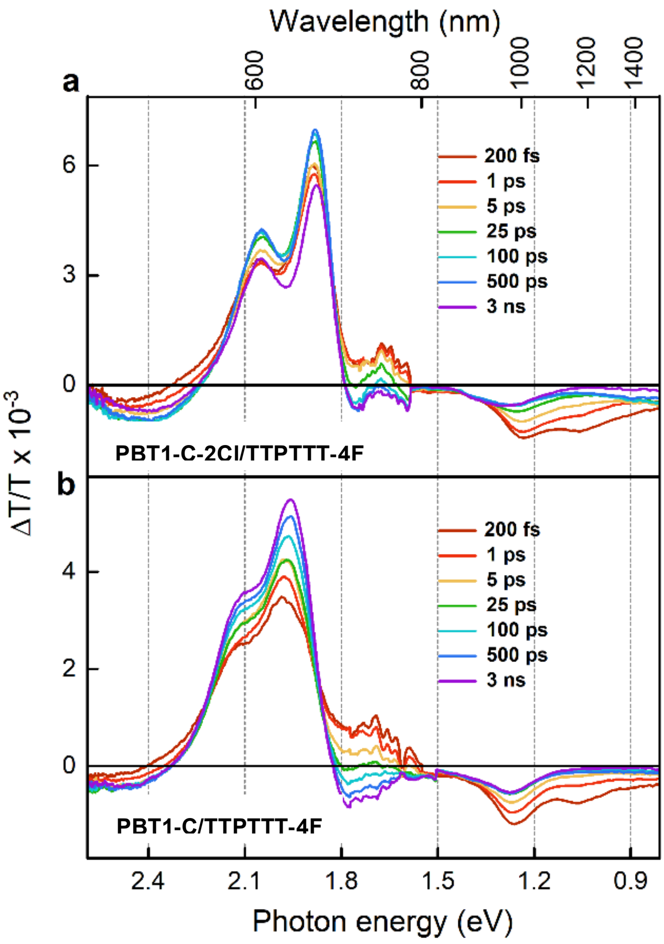

Figure S17. Series of transient absorption spectra of a) PBT1-C-2Cl/TTPTTT-4F and b) PBT1-C/TTPTTT-4F blends following excitation at 550 nm, at pump fluences of 2 μJ/cm^2 and 3 μJ/cm^2 respectively.

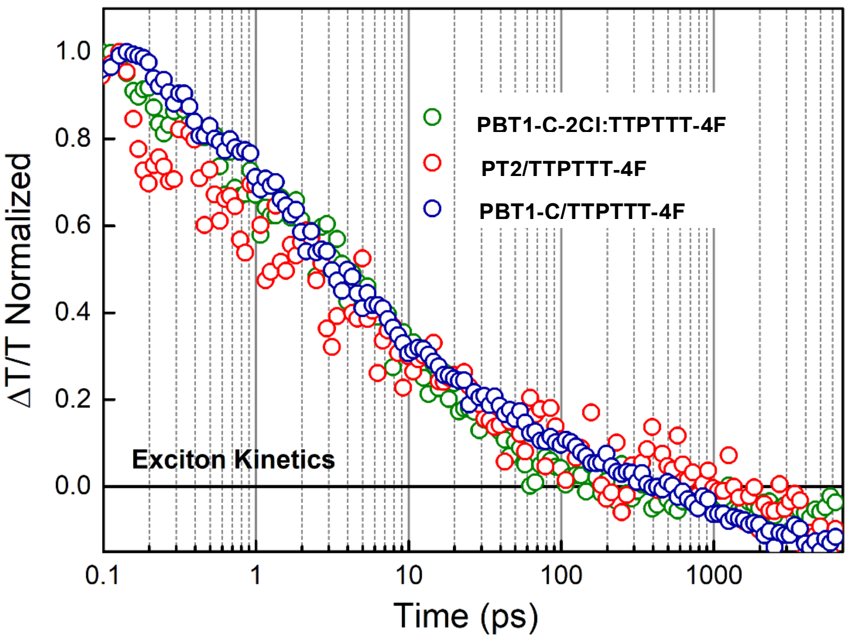

Figure S18. Normalized exciton kinetics of PBT1-C-2Cl:TTPTTT-4F, PT2:TTPTTT-4F, and PBT1-C:TTPTTT-4F blend films following an excitation at 550 nm at pump fluences of 2 μJ/cm^2, 3 μJ/cm^2 and 3 μJ/cm^2 respectively.

References

[1] T. Liu, L. Huo, S. Chandrabose, K. Chen, G. Han, F. Qi, X. Meng, D. Xie, W. Ma, Y. Yi, J. M. Hodgkiss, F. Liu, J. Wang, C. Yang, Y. Sun, *Adv. Mater.* **2018**, *30*, 1707353.

[2] L. Ye, Y. Xie, K. Weng, H. S. Ryu, C. Li, Y. Cai, H. Fu, D. Wei, H. Y. Woo, S. Tan, Y. Sun, *Nano Energy* **2019**, *58*, 220.

[3] K. Weng, L. Ye, L. Zhu, J. Xu, J. Zhou, X. Feng, G. Lu, S. Tan, F. Liu,Y. Sun, *Nat. Commun.* **2020**, *11*, 2855

[4] J. Song, C. Li, L. Zhu, J. Guo, J. Xu, X. Zhang, K. Weng, K. Zhang, J. Min, X. Hao, Y. Zhang, F. Liu, Y. Sun, *Adv. Mater.* **2019**, *31*, 1905645.

[5] D. Nowak, W. Morrison, H. K. Wickramasinghe, J. Jahng, E. O. Potma, L. Wan, R. Ruiz, T. R. Albrecht, K. Schmidt, J. Frommer, D. P. Sanders, S. Park, *Sci. Adv.* **2016**, *2*, e1501571.

[6] J. Jahng, E. O. Potma, and E. S. Lee, *Anal. Chem.* **2018**, *90,* 11054.
